# A meta-analysis of the meaning in life and suicidal ideation based on Chinese samples

**DOI:** 10.3389/fpsyg.2025.1610351

**Published:** 2025-08-01

**Authors:** Xinguo Yang, Lei Xiao, Yuanmei Lan, Jingdan Xue, Yunxiao He

**Affiliations:** ^1^School of Marxism, Guangxi University, Nanning, China; ^2^School of Public Policy and Management, Guangxi University, Nanning, China

**Keywords:** meaning in life, suicidal ideation, meta-analysis, Chinese context, suicide prevention

## Abstract

Previous research has examined the link between meaning in life and suicidal ideation across cultures, highlighting cultural background as a moderator. However, few studies focus on single cultural contexts. To address this gap within China, this meta-analysis investigates the association between meaning in life and suicidal ideation, specifically examining moderators including regional economic differences, measurement tools for suicidal ideation, and the impact of public health emergency (e.g., COVID-19). Results show a negative correlation between meaning in life and suicidal ideation [*r* = −0.387, 95% CI (−0.425, −0.344)]. Subgroup analyses revealed stronger effects in undeveloped regions, when using specific measurement tools, and during public health emergency. Unlike previous studies, gender did not significantly moderate this relationship. These findings confirm that enhancing meaning in life may be a key protective factor against suicidal ideation in China, especially in undeveloped regions and during crises. Future interventions should develop tailored strategies to strengthen meaning in life, prioritizing economic inequality and crisis resilience.

## 1 Introduction

Suicide is a serious public health problem worldwide (World Health Organization, 2022). According to statistics from the National Health Commission, the suicide rates among urban populations of China aged 10–15, 15–20, and 20–25 have increased from 0.96:100,000, 1.40:100,000, and 1.59:100,000 in 2017 to 1.70:100,000, 3.34:100,000, and 3.45:100,000 in 2021, more than doubling on average. Concurrently, the suicide rates among adolescents in rural areas of China have also reached high levels at 1.66:100,000, 3.65:100,000, and 3.66:100,000 (National Health Commission, 2023). The abnormal mortality rate of college students was 4.94:100,000, and the suicide rate was 2.37:100,000. Among the five types of abnormal mortality among college students, the proportion of suicide accounted for 47.2% (Yang and Li, 2015). Suicidal ideation (hereafter, SI) is a part of suicide and can evolve into further suicidal behaviors (Hubers et al., 2018). Studies indicate that suicidal ideation is prevalent across various populations: data from psychological assistance hotlines show that the proportion of first-time callers with SI was 30.9% in Henan Province and 47.2% in Beijing (Liu et al., 2025; Wang et al., 2022). Studies on student populations reveal that the overall prevalence rate of SI among Chinese students is 13.6%, with rates of 23.5% among middle school students, 10.72% among university students, and 8.8% among graduate students (Yu and Wang, 2024; Li et al., 2014). How to intervene and reduce SI is a key component of suicide prevention (Arria et al., 2009; Harris and Barraclough, 1997).

A robust predictor of SI is the loss of MIL (Harlow et al., 1986). While empirical evidence generally supports a negative link between MIL and SI (Lutzman et al., 2021), inconsistencies exist across studies regarding the strength of this association (Luo et al., 2018; Yang et al., 2022). Furthermore, it remains unclear whether the relationship between MIL and SI is influenced by other factors. Some studies suggest that gender may play a moderating role in this relationship, as men and women differ in their attribution of life meaning, while other studies have not found significant gender effects on this relationship (Morgan, 2013; Schulenberg et al., 2016; Aviad-Wilchek and Ne’eman-Haviv, 2018; Kiang and Fuligni, 2010). Regarding age differences, divergent perspectives also exist: one body of research suggests that individuals’ developmental stages lead to variations in the relationship between MIL and SI (McLean et al., 2010; Tavernier and Willoughby, 2012); while other studies propose that this association demonstrates relative stability across different age groups (Bamonti et al., 2016; Reker and Fry, 2003). The selection of measurement tools may also significantly impact the results. Existing researches have found that there were significant differences in measurement outcomes between SI tools that are not fully adapted to the local context and those developed based on the local culture (He et al., 2023). This difference may arise because the design of these tools failed to adequately consider the cultural background, social characteristics, and psychological traits of the region, thereby limiting their applicability and effectiveness. Economic conditions may also moderate the effect of life meaning on suicidal ideation by influencing individuals’ living conditions and goal pursuits, regional economic recession has been associated with higher suicide risk (Mann and Metts, 2017).

A recent cross-cultural meta-analysis (Li et al., 2024) examined MIL-SI links in adolescents, identifying socioeconomic and cultural factors as moderators but not gender or age. However, critical gaps persist, specifically for understanding this relationship within China’s unique context: Li et al.’s study included very few collectivist samples (only 3 out of 18); It did not assess methodological moderators like SI measurement tools, acknowledged as a limitation; And it omitted the profound impact of recent public health emergency (e.g., COVID-19), which had distinct psychological consequences in China due to specific national policies and prolonged measures (Zhao et al., 2024; Chen et al., 2024).

Crucially, established moderators may operate differently within China: Cultural expectations around gender roles may uniquely shape coping strategies related to MIL loss (Zhang D. et al., 2022; Zhang J. et al., 2022). Significant urban–rural economic disparities influence resource access and mental health support, potentially intensifying the MIL-SI link in disadvantaged regions (Lin et al., 2024; Wei et al., 2018). Furthermore, the validity of Western-developed SI measurement tools within China’s distinct sociocultural and psychological landscape is a significant concern (He et al., 2023; Zhong et al., 2024).

To further examine the relationship between SI and MIL in the Chinese context, this study intends to employ the meta-analysis method. Meta-analyses can draw a comprehensive conclusion by analyzing relevant quantitative literature data and calculating combined effect sizes (Lipsey and Wilson, 2001). In recent years, scholars in China have also conducted a plethora of empirical studies on SI and MIL, which has provided a robust empirical foundation for meta-analysis. Therefore, this study will conduct a Meta-analysis of 39 empirical studies on the relationship between MIL and SI in the Chinese context to achieve the following innovations: (1) To systematically clarify the relationship between MIL and SI in the Chinese context, and to compare it with study in a cross-cultural context, thereby obtaining conclusion about the relationship between MIL and SI in collectivism countries. (2) To examine whether the moderators proposed in a cross-cultural study work in the Chinese context. (3) To examine two new moderators, SI measurement tools and public health emergency, which were proposed in the Chinese context. This study explains the mechanisms of the moderators on the relationship between MIL and SI to provide reference for research on the MIL-SI relationship in the Chinese context.

### 1.1 The research questions and hypotheses of the present study

In order to integrate the disparate findings from different studies in the Chinese context, this study employs a meta-analysis to synthesize the literature and examine the association between MIL and SI in the Chinese context, as well as the variables that moderate this association. This study will address the following two issues:

(1) The association between MIL and SI in the Chinese context (positive, negative, or no correlation)?
(2) Do the following variables moderate this association: gender, economy, tools, and public health emergency?

### 1.2 The relationship between meaning in life and suicidal ideation in the Chinese context

Empirical studies reveal that the phenomenon of suicide in the Chinese context presents a socio-cultural pattern distinctly different from Western societies. This difference is first reflected in the impact mechanisms of individualism and collectivism on suicidal behavior. In individualism countries, individuals are encouraged to pursue autonomy and independence. However, when facing setbacks or adversity, individuals in this cultural background are more prone to self-attribution bias, blaming their failures on their lack of ability, thereby exacerbating psychological burdens. At the same time, the relatively loose social support network further amplifies the risk of SI (Cross and Madson, 1997; Antonucci and Akiyama, 1987). In contrast, in collectivism countries like China, families and society often provide a tighter support system when individuals face difficulties, thus buffering against the psychological stress brought about by adversity to some extent (Markus and Kitayama, 1991). This cultural differentiation also extends to the level of psychopathology. Western studies have found that the prevalence of mental disorders among those who died by suicide is significantly higher (Cavanagh et al., 2003), whereas suicide cases in China exhibit lower associations with mental illness (Phillips et al., 2002; Zhang, 2010). One possible explanation is that the phenomenon of suicide in China is more driven by socio-cultural factors rather than purely pathological reasons. Additionally, China exhibits unique gender difference characteristics, having been one of the few collectivism countries where the female suicide rate was higher than that of males (Phillips et al., 2002). This phenomenon echoes contemporary study’s findings of an imbalance in the proportion of female SI (Lu et al., 2020). From a cultural perspective, this can be attributed to the lower socio-economic status of rural women in traditional agricultural societies, especially the oppression and inequality they face in marriage and family, which may subject them to greater psychological stress. The easy accessibility of highly lethal means such as pesticides further increases the risk of female suicide (Chen et al., 2017). With a series of effective intervention measures implemented by the government in rural areas in recent years, the suicide rate of rural women has shown a significant downward trend (Zhang D. et al., 2022; Zhang J. et al., 2022). On the other hand, men, due to the demands of traditional gender norms for a strong image, often choose to conceal their emotions rather than seek help when facing psychological crises. This solidification of gender roles not only exacerbates the different responses of the sexes in dealing with psychological stress but also reveals the profound impact of gender inequality in the process of modernization, making the phenomenon of suicide in the Chinese context exhibit deep cultural specificity.

The construction of MIL in the Chinese context manifests unique cultural attributes characterized by limited religious adherence (Stark and Liu, 2011). In the absence of institutionalized spiritual frameworks (Schlegel et al., 2009; Oishi and Diener, 2014), individuals engage with indigenous philosophical systems—Confucianism, Taoism, and Buddhism—forming a tripartite value matrix that reconstitutes MIL through secular existential paradigms (Redding, 2013). This cultural particularity emerges from the dialectical synthesis of autochthonous wisdom traditions and assimilated religious thought, establishing non-theistic pathways for ontological significance attribution distinct from Western MIL systems.

Based on the aforementioned studies, we believe that the relationship between individuals’ SI and MIL in the Chinese context may be similar to the findings in cross-cultural research, but the underlying influencing factors could be significantly different. Therefore, we propose the following hypothesis:

*H1*: MIL and SI will demonstrate a negative correlation in the Chinese context.

### 1.3 Moderator

**Figure 1:**
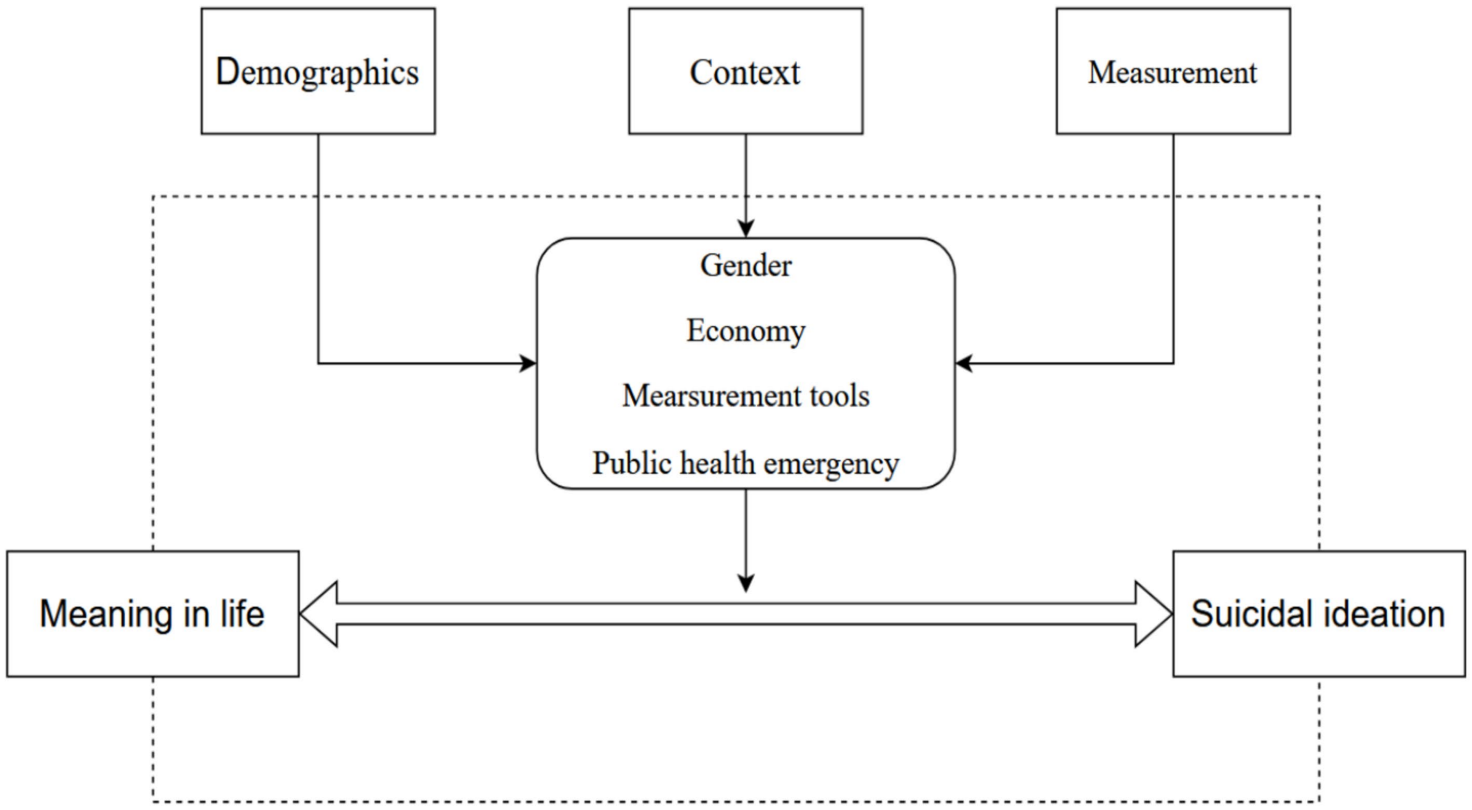
The relationship between MIL and SI with potential moderators (Theoretical Framework).

In the existing meta-analysis study on MIL and SI, four moderating variables were selected: gender, age, economic factors, and culture. However, due to the fact that not all samples included in our study reported age information, and considering the limitations of the sample—most of which were student populations—age was not chosen as a moderating variable in this study. By referencing the findings of existing study and considering the unique aspects of conducting MIL and SI research in the Chinese context, we propose two new moderators, tools and public health emergency, based on the two existing moderators, gender and economy. Four moderators from three aspects (demographics, context, and measurement) were identified. All details are shown in Figure 1.

#### 1.3.1 Gender

The gender differences in suicide risk present a unique picture in the Chinese cultural context, diverging significantly from the global norm. The deep-rooted origins of this phenomenon are embedded in the family order and gender role norms within Chinese culture. Although global epidemiological data show that male suicide rates generally exceed those of females—a phenomenon known as the “gender paradox” (Canetto and Sakinofsky, 1998)—China has long been a notable exception, with historical data showing higher female suicide rates than males. While this gap has narrowed over the past few decades, it still markedly differs from Western patterns (Zhang D. et al., 2022; Zhang J. et al., 2022). This “Chinese exception” may result from the interplay of multiple factors.

Firstly, under the influence of Confucian culture, family power structures and the pressures placed on women play a critical role. Confucian traditions emphasize hierarchical order and role obligations within the family, imposing strict norms on women. This often places women in a subordinate position within the power structure, leaving them vulnerable when facing intense marital conflicts, fertility pressures, or unmet societal expectations. Lacking effective buffering mechanisms and support resources, their suicide risk is significantly heightened (Zhang et al., 2011a).

Secondly, the “protective” and “stigmatizing” aspects of male roles offer insight. Patriarchal ethics grant men higher status and authority while simultaneously providing them with stronger identification with family responsibilities and access to social support networks, thus offering some degree of protection. More importantly, the Chinese cultural interpretation of suicide behavior contrasts sharply with that of the West. In Western contexts, suicide is often linked to notions of “masculinity” (Canetto, 2008), whereas Chinese culture tends to view it as an act of “weakness lacking masculinity” or “escaping responsibility.” This strong de-masculinization stigma creates significant cultural and psychological barriers for male suicides (Jia and Zhang, 2017).

Thirdly, meaning conflict and “the weapons of the weak” further explain the high vulnerability of Chinese women. Women are often caught between the conflicting demands of traditional roles and modern personal values. Perceived gender inequality has been shown to be positively correlated with suicidal ideation (Zhang et al., 2019). In extreme situations, suicide is sometimes seen by some women as a desperate means to express despair, protest injustice, or seek liberation (Zhang, 2010). This culturally embedded cognitive framework and stress mechanism continue to influence contemporary suicide ideation reports: comparative studies indicate that even amid a more balanced gender ratio, Chinese women still report significantly higher levels of suicidal ideation than men (Zhang D. et al., 2022; Zhang J. et al., 2022).

Therefore, we propose the hypothesis 2:

*H2*: In the Chinese context, the moderating effect of gender on the MIL - SI association will be significant, and the correlation between MIL and SI in female samples is stronger than that in male samples.

#### 1.3.2 Economy

The economic conditions of the surveyed regions may also profoundly influence the association between MIL and SI. Early research indicates that economic environments not only shape individuals’ daily living standards but also, by affecting resource accessibility and opportunity structures, influence their pathways to fulfilling personal aspirations and the foundational construction of MIL (Pezirkianidis et al., 2016). Within China’s unique sociocultural context, this economic influence intertwines with rapid urbanization, modern cultural impacts, and the urban–rural dual structure, presenting a complex landscape.

In developed regions, modern culture spurred by reform and opening-up emphasizes personal achievement and market competition, leading individuals to anchor MIL more frequently in career advancement or material success. Conversely, in undeveloped regions experiencing delayed urbanization or cultural disruption, traditional rural ethics—though challenged by modernization waves—remains the core framework for MIL construction among many (Huang et al., 2023).

This economic-cultural embeddedness renders the impact of economic fluctuations on MIL potentially more acute in underdeveloped regions: when economic development lags or faces setbacks, it signifies not only material deprivation but also the potential failure to fulfill culturally rooted “life tasks,” thereby profoundly destabilizing individuals’ sense of meaning.

Therefore, we propose the hypothesis 3:

*H3*: In the Chinese context, the moderating effect of the economy on the MIL - SI association will be significant, and the correlation between MIL and SI in undeveloped areas samples is stronger than that in developed areas samples.

#### 1.3.3 Tool

Compared to measurement tools for MIL, instruments assessing SI exhibit greater diversity, with different tools potentially emphasizing distinct conceptual dimensions of SI such as intensity, frequency, or specific content. Beck’s Suicide Ideation Scale (SSI) focuses primarily on evaluating ideation intensity, whereas Osman’s revised Suicide Behaviors Questionnaire-Revised (SBQ-R) emphasizes ideation frequency. The validity of these instruments is highly dependent on their developmental cultural context. Given Western countries’ pioneering advantage in SI research, many Chinese studies have directly translated and used these original tools. However, the core conceptual frameworks of Western scales—including their emphasis on individual autonomy, religious beliefs, or specific suicide methods—may fundamentally misalign with SI expression patterns within China’s collectivist cultural context (He et al., 2023). Specifically, this cultural mismatch manifests in two key aspects.

First, Western instruments may overlook SI triggers critically significant in Chinese culture, such as intense “family shame” (from unfulfilled filial duties or ancestral dishonor), despair following failed major “life tasks,” or “altruistic relief” thoughts during intense family conflicts (Ma et al., 2022). Second, influenced by Confucian principles like avoiding public disclosure of family shame or the culture of endurance, Chinese individuals often prefer somatizing distress or using metaphorical language to report SI (e.g., “life feels meaningless” or “being a burden to the family”) rather than explicit “wanting to die” statements. Consequently, Western scales relying on direct phrasing may underestimate true risk (Lu et al., 2020). Therefore, researchers emphasize systematically adapting or redeveloping tools based on China’s unique sociocultural characteristics (Xia et al., 2002). Critically, such cultural adaptation has proven essential, as meta-analytic evidence confirms that SI measurement choice significantly moderates SI’s correlations with other variables (He et al., 2023). We thus reasonably infer that this culturally induced measurement “noise” similarly interferes with—or even distorts—capturing the true MIL-SI relationship.

Therefore, we propose the hypothesis 4:

*H4*: In the Chinese context, the moderating effect of measurement tools of SI (tools) in the MIL - SI association will be significant, and the correlation between MIL and SI in using Chinese-revised tools samples is stronger than that in using foreign original tools samples.

#### 1.3.4 Public health emergency

The impact of public health emergency on the relationship between MIL and SI may exhibit significant specificity in terms of its pathways and intensity within China’s unique collectivist cultural context, warranting further investigation. The novel coronavirus strain SARS-CoV-2 was first identified at the end of 2019 and rapidly spread globally the following year, causing a worldwide pandemic (COVID-19). This health crisis not only disrupted individuals’ existential stability through universal stressors such as prolonged lockdowns, health anxiety, and social isolation (Trzebiński et al., 2020; Koştu et al., 2024), but also interacted complexly with cultural psychological factors in the Chinese context, including traditional sentiments of family and country, socialist culture’s sense of community, and trust in government.

Firstly, there is the “meaning-making” transformation of stressors: Compared to individualism countries that emphasize individual freedom restrictions and rights loss due to the pandemic, China’s collectivist values might lead some individuals to internalize strict epidemic prevention measures as a collective responsibility and act of dedication toward “protecting families and society,” even endowing it with a lofty sense of safeguarding “national security.” This, to some extent, helps buffer the loss of MIL (Aytekin et al., 2024).

Secondly, the strengthening of community bonds and the spirit of “solidarity in adversity”: The cultural tradition of “when one place is in trouble, help comes from all sides” has been widely activated during the pandemic. Neighborhood mutual aid, community-organized volunteer activities, and the state’s high emphasis on livelihood security have reinforced the sense of connection and belonging among social members, becoming an important cultural resource against isolation and maintaining MIL (Prati and Mancini, 2021).

Finally, the construction of institutional trust and the significance of “security provision”: The strong mobilization capabilities and resource investment demonstrated by the Chinese government under the principle of “people first, life first” might provide citizens with a deep sense of “security provision” and “order assurance.” This trust in institutional protective capacity can be transformed into a collective-level support for meaningfulness, partially offsetting individual panic in the face of uncertainty (Ma and Lyu, 2021).

However, the accumulation of long-term stress and the tension of cultural expectations should not be overlooked: Concerns about family livelihoods, the impact of strict quarantine policies on traditional family reunions, and the practical difficulties in fulfilling filial piety or parenting responsibilities may trigger unique “meaning conflicts” and ethical anxieties within the Chinese cultural context, becoming potential sources of SI risk (Zhao et al., 2024; Hao et al., 2024). Therefore, the impact of the pandemic on the MIL-SI relationship among the Chinese population could be a double-edged sword—providing protection through activating collectivism cultural resources and institutional trust while exacerbating risks due to the obstruction of core cultural needs.

Therefore, we propose the hypothesis 5:

*H5*: In the Chinese context, the moderating effect of public health emergency on the meaning-in-life—SI association will be significant. The correlation between meaning-in-life and SI in samples after the pandemic is stronger than that in samples before the pandemic.

**Figure 2:**
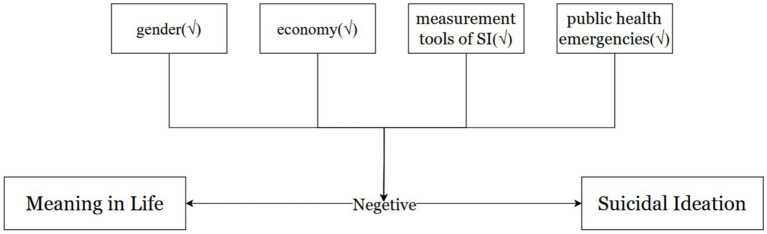
Hypotheses of the study. √ indicates a significant moderating effect.

All hypotheses are shown in Figure 2.

## 2 Method

### 2.1 Literature search

#### 2.1.1 Search strategy

A comprehensive literature search was performed in primary Chinese academic repositories (including CNKI, Wanfang Data, and VIP Journals Database) alongside international databases (encompassing Wiley, Science Direct, ProQuest, Web of Science, and EBSCO). This investigation systematically integrated terminology related to meaning in life (specifically “meaning in life,” “life meaning,” “purpose in life,” “life purpose,” and “psychological coherence”) with suicide-related concepts (“suicidal ideation” and “suicide intention”) within the designated search parameters of titles, abstracts, and keywords. To address dissemination bias, supplementary search efforts incorporated general web search tools and bibliographic citations, encompassing pertinent review articles.

**Figure 3:**
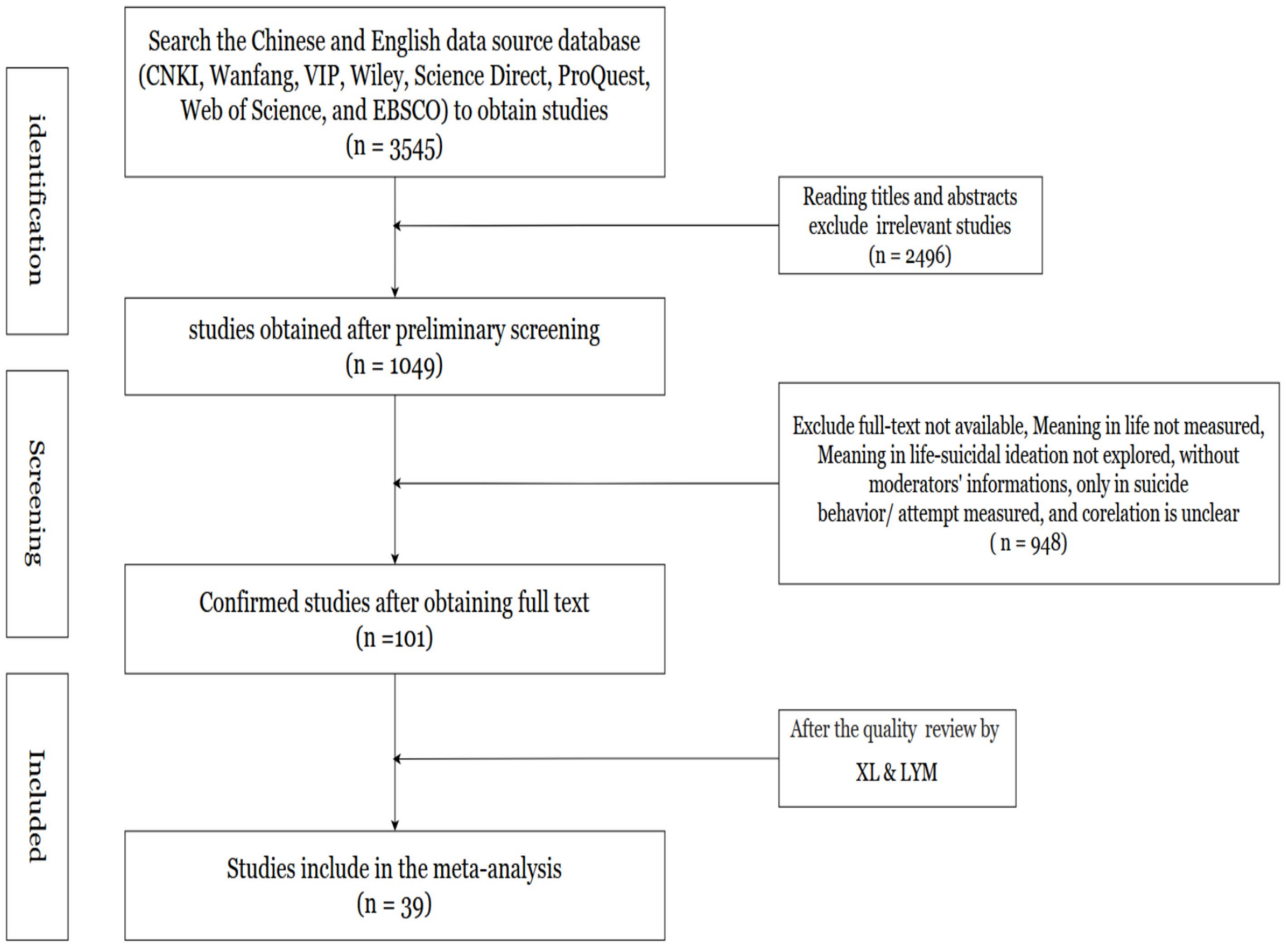
PRISMA diagram of the search history.

The research strictly adhered to PRISMA (Page et al., 2021) reporting standards, with methodological implementation depicted in Figure 3. This involved dual-phase screening of article headings and summaries for thematic congruence, followed by in-depth assessment of candidate publications against predefined eligibility thresholds. Furthermore, bibliographies of complete-text publications were systematically examined to identify pertinent research, with dual-reviewer verification (XL and LYM) implemented to maintain selection validity.

#### 2.1.2 Inclusion criteria

The present study adopted the following inclusion criteria: (1) an empirical paper; (2) the study reported the correlation coefficient between MIL and SI or provided data that could be converted into a correlation coefficient; (3) The samples between studies were independent. In instances of overlap or repetition of samples between studies, the study with more detailed data or a larger sample size was selected. (4) The study reported the measurement tools for SI.

Papers were excluded if: (1) the study incorporated content about SI yet lacked data on the subject; (2) it did not incorporate measurement of MIL.

#### 2.1.3 Data extraction

The study collected information on four domains: (1) publication information: author and year of publication, journal; (2) demographic information: gender ratio of the participants; (3) The study characteristics: time of study conduct, region, and the measurement tools; (4) The variables: sample size (N) and the correlation coefficient (r) between MIL and SI. In studies that reported correlation coefficients in two distinct dimensions, the correlation coefficient was obtained by calculating the weighted average of the two. The third author (XJD) independently verified all collected datasets, and discrepancies were eliminated by achieving consensus through team consultations.

### 2.2 Coding procedures

The gender variable was achieved by calculating the female proportion reported in the sample. The variable of economy was referenced based on the division by Su et al. (2021) into developed and undeveloped regions (developed regions include the Bohai Rim, Pearl River Delta, Yangtze River Delta, Harbin-Changchun-Shenyang area, Southern Jiangsu urban agglomeration, Zhengzhou metropolitan area, Wuhan-Changsha-Nanchang-Hefei urban agglomeration, Chengdu-Chongqing urban agglomeration, and the Xi’an; undeveloped regions refer to areas outside of the aforementioned regions. For studies that cover multiple regions, we categorize them as cross-regional). Public health emergency was based on the survey time reported in the sample, with 1 February 2020 designated as the reference point. Studies that conducted investigations before this date were categorized as pre-pandemic, while those conducted after this date were categorized as post-pandemic. The SI measurement tools utilized in the studies encompassed by this study were categorized based on the sample reports into two distinct types: Chinese-revised tools and foreign original tools. Foreign original tools refer to tools developed by foreign scholars that are directly translated into Chinese for use in studies without undergoing cultural adaptation or localization. Among them, the Suicidal Ideation Self-Rating Scale (SIOSS) was compiled by Chinese scholars. The Chinese version of the Suicidal Behavior Questionnaire-Revised (SBQ-R-C), the Chinese version of the Piers-Harris Children’s Self-Concept Scale-Suicidal Ideation Subscale (PANSI-C), the Chinese version of the Beck Scale for Suicidal Ideation (BSI-C), and the Suicidal Ideation and Behavior Questionnaire (SIBQ) were translated and revised by Chinese scholars. The remaining scales are foreign original tools.

### 2.3 Statistical analysis

To investigate the meta-analysis results of the relationship between MIL and SI, this study used R statistical software (R Core Team, 2022) to conduct heterogeneity tests, publication bias tests, main effect tests, and moderator effect tests. All data analyses were executed through R statistical software (R Core Team, 2022), with effect size computations implemented via the metaphor package (Viechtbauer, 2010; Harrer et al., 2021), and visualization of funnel plots achieved using the meta package (Assink and Wibbelink, 2016; Balduzzi et al., 2019; Harrer et al., 2021). The heterogeneity tests in the meta-analysis reported values such as *Q, df(q), p,* and *I*^2^. Publication bias is a critical criterion for evaluating the accuracy of the results, the fail-safe N represents the minimum number of unpublished studies that would need to be considered to reasonably exclude publication bias in the meta-analysis (Rosenthal, 1979). When the fail-safe coefficient exceeds 5K+10, it indicates that the research results are less affected by publication bias and are more stable. The main effect reported values include *K1*, correlation coefficient *r*, *Z*-value, *p*-value and Tau Squared. The moderator tests examine the magnitude of moderators’ influence on the results, reporting the coefficients of the moderators, confidence intervals, and credible intervals.

## 3 Results

### 3.1 Characteristics of the original literature included

The characteristics of the literature are presented in Table 1. 39 articles were analyzed, 23 written in Chinese and 16 written in English. The publication dates of the articles ranged from 2008 to 2024. We extracted 39 independent samples from 39 articles, comprising 69,028 participants. Among these samples, 8 showed a male majority compared to female representation, whereas 31 demonstrated a predominant female composition over males. Twenty-five samples were conducted before 1 February 2020, while 14 were conducted subsequently. Ten cross-regional studies were conducted; 21 were conducted in developed regions, and eight were surveyed in undeveloped regions.

**Table 1:** Characteristics of studies included in the meta-analysis.

Name	Type	Sample	Women (%)	Tools	Area	Time
Zhong et al. (2024)	J	3,132	75	SBQ-R-C (Chinese-revise)	Guangzhou	2023
Yang et al. (2022)	J	4,515	49.8	DSI-SS (foreign original)	Hunan, Guangdong, Jiangxi, Anhui	2022.3–2022.7
Liu et al. (2025)	J	16,508	52.2	DSI-SS (foreign original)	Hunan	2022
Zhang et al. (2013)	J	1,352	51.38	PANSI-C (Chinese-revise)	Beijing, Shandong, Sichuan, Hunan, Guangxi	2013
Gen and Zhang (2008)	J	830	48.3	SIBQ (Chinese-revise)	Guangzhou	2008
Zhang Y. et al. (2021)	J	1847	59.94	BSI-C (Chinese-revise)	Langfang	2020.5–2020.10
Luo et al. (2018)	J	420	76.4	BSI-C (Chinese-revise)	Guangzhou, Foshan	2018
Zhao et al. (2013)	J	1,310	65.06	SBQ-R (foreign original)	Guangzhou	2011.10–2011.11
Shi (2018)	D	410	44.39	PANSI-C (Chinese-revise)	Gansu	2018
Cui and Zhang (2024)	J	444	48.2	SIOSS (Chinese-revise)	Liaoning	2024
Zhang et al. (2019)	J	1,148	46.1	BSI-C (Chinese-revise)	Guangxi	2019
Yuan (2019)	D	1,432	59.91	PANSI-C (Chinese-revise)	Liaoning	2019
Li (2021)	D	500	52.4	PANSI-C (Chinese-revise)	Gansu	2020
Zhang et al. (2024)	D	749	48.87	SIOSS (Chinese-revise)	Hubei	2023
Zeng et al. (2018)	J	2,787	64.9	SBQ-R (foreign original)	Guangzhou	2015.11
Zhang J. et al. (2022)	J	671	31.9	PANSI-C (Chinese-revise)	Beijing	2020.9
Chen (2012)	D	262	74.4	SIOSS (Chinese-revise)	Quanzhou	2011
Zhang et al. (2024)	J	603	34.6	PANSI-C (Chinese-revise)	Beijing	2023
Ha et al. (2022)	J	5,557	67.6	SBQ-R (foreign original)	Shanxi, Gansu, Ningxia, Xinjiang	2022
Qin et al. (2021)	J	1,574	76.6	SBQ-R (foreign original)	Shandong	2017
Chen (2012)	J	1,017	67.6	SIOSS (Chinese-revise)	Fujian	2012
Gan (2019)	D	654	59.2	SIOSS (Chinese-revise)	Nanjing	2019
Xiang et al. (2016)	J	646	61.7	SIOSS (Chinese-revise)	Guangdong	2016
Yuan and Wang (2021)	J	90	63.33	SIOSS (Chinese-revise)	Shandong	2017.6
Zhang Y. L. et al. (2021)	J	633	59.72	PANSI-C (Chinese-revise)	Shenyang	2020.7
Wang and Wang (2023)	J	767	57.9	SBQ-R-C (Chinese-revise)	Zhengzhou	2020.2–2020.3
Tan et al. (2018)	J	6,165	50.1	BSI-C (Chinese-revise)	Eastern is	2017
Yu and Wang (2024)	J	1,472	84.6	PANSI (foreign original)	Chongqing	2021
Zhang et al. (2024)	J	505	100	BSI-C (Chinese-revise)	Hubei, Hunan	2022.3–2022.10
Lew et al. (2020)	J	2074	66	SBQ-R (foreign original)	Shandong	2020
Zhao et al. (2013)	J	1,390	53.6	SBQ-R-C (Chinese-revise)	nationwide	2012
Liu et al. (2021)	J	687	55	SIOSS (Chinese-revise)	nationwide	2018
Fong et al. (2023)	J	1,472	51.7	PHQ-4 (foreign original)	Hong kong	2021
Li et al. (2024)	J	2,615	61	SBQ-R-C (Chinese-revise)	Guangzhou	2020.3
Lin et al. (2022)	J	276	64.8	PANSI (foreign original)	Taiwan	2019
Liu et al. (2021)	J	687	55	SIOSS (Chinese-revise)	nationwide	2019
Zhang (2019)	J	931	57.2	PANSI (foreign original)	Shanghai	2017
Liu et al. (2021)	J	665	53.23	SIOSS (Chinese-revise)	Beijing, Shandong, Sichuan, Shanxi	2020.2
Feng (2023)	D	231	63.2	BSI-C (Chinese-revise)	Nanchang	2022.5–2022.12

Name = Article Name; Type = Article Type, J for journal articles, D for dissertations; Sample = Sample Size; Women% = Proportion of females in the sample; Tools = SI measurement tools; Area = Region of survey conduct; Time = Time of survey conduct.

### 3.2 Heterogeneity test

The Q-test revealed substantial heterogeneity across effect size estimates (*Q* = 1390.391, *p* < 0.001), with an overall *I*^2^ value of 97.267% (exceeding the 75% critical threshold) confirming significant variability in the primary research outcomes as documented in Table 2. This statistical evidence necessitated subsequent moderator analysis to systematically identify potential sources of divergence within the study findings.

**Table 2:** Heterogeneity test result.

Q	df(q)	*p*	*I* ^2^
1390.391	38	0.000	97.267

### 3.3 Publication bias test

**Figure 4:**
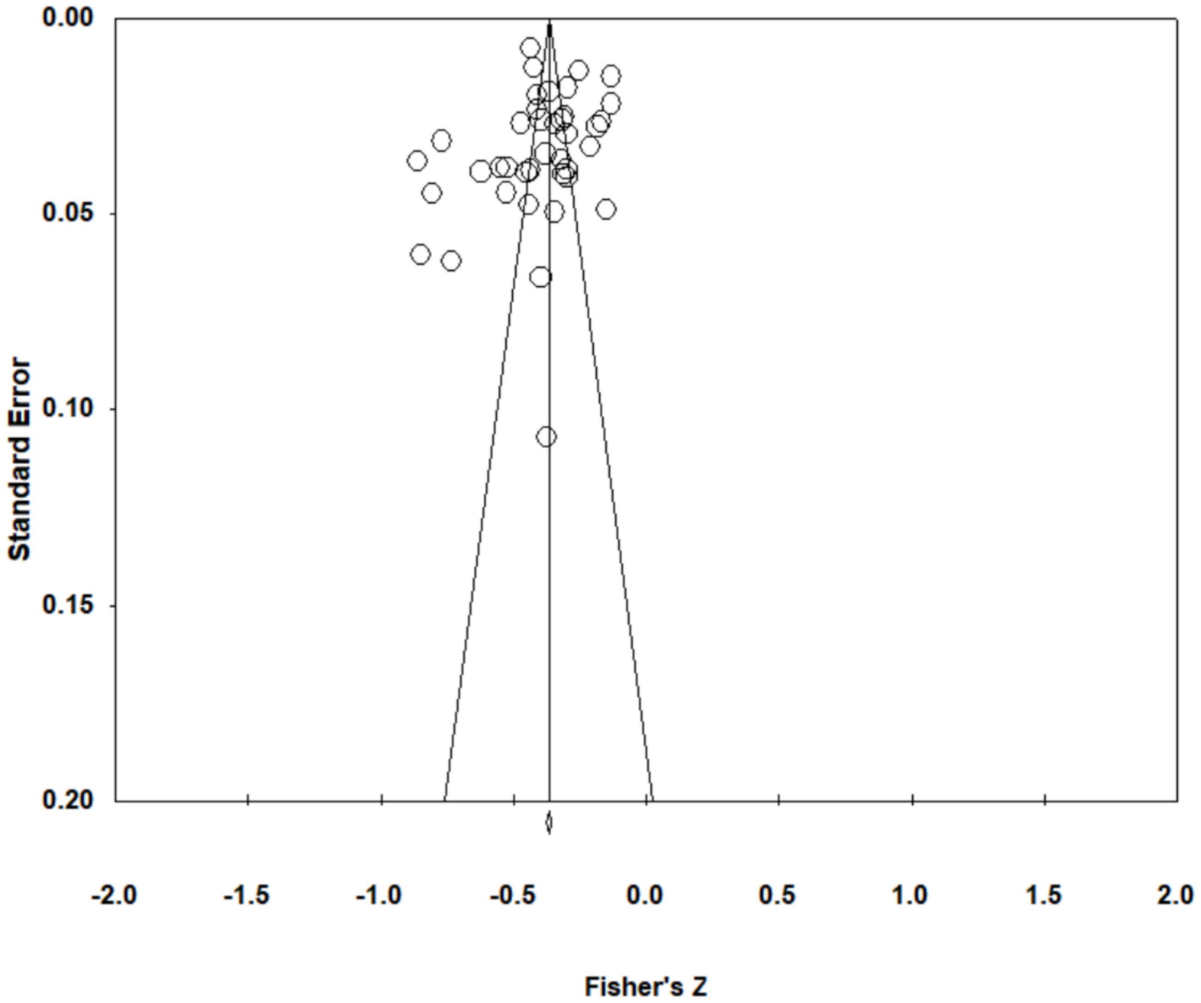
Funnel plot for publication bias test.

The Classic Fail-Safe N test *Z*-value is −86.058. The insecurity coefficient, Nfs = 5,151, is more significant than 5 K + 10 = 205. Multiple research investigations corroborate the favorable outcomes observed, suggesting a minimal likelihood of publication bias (Refer to Table 3). The visual representation of the funnel plot is depicted in Figure 4.

**Table 3:** Classic fail-safe N.

Z-value for observed studies	−86.05883
The *p*-value for observed studies	0.00000
Alpha	0.05000
T ails	2.00000
Z for alpha	1.95996
Number of observed studies	39.00000
Number of missing studies that would bring *p*-value to > alpha	5151.00000

### 3.4 Main effect

A statistically significant inverse association was identified between MIL and SI [*r* = −0.387, 95% CI (−0.425,-0.344)], as presented in Table 4. Based on the classification framework established by Ellis (2010), this correlation magnitude was categorized as a moderate-strength negative relationship.

**Table 4:** Main effects of the relationship between MIL and SI.

K1*	*r* (95% CI)	Z-value	*p*-value	Tau Squared
39	−0.387 (−0.425, −0.344)	−16.753	0.000	0.022

*K1 = Number of studies.

### 3.5 Moderating effects test

**Figure 5:**
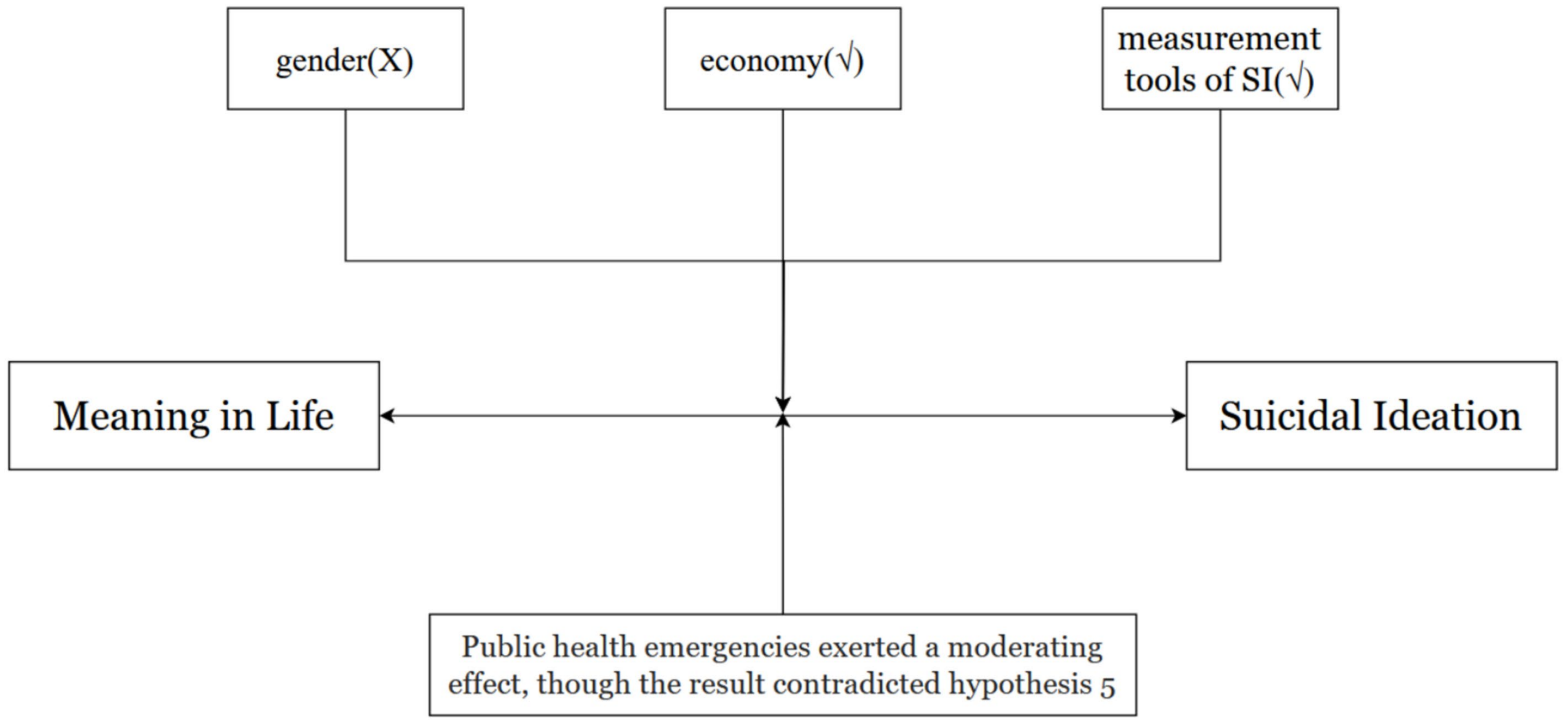
The result of moderating effect test.

The 39 samples were integrated into meta-regression modeling to explore the potential moderating effects of gender, economy, tools, and public health emergency. The test results of the moderating effect are shown in Figure 5.

#### 3.5.1 Gender

The gender ratio of the samples was derived from self-reported data. For samples that did not directly report the gender proportion, the ratio was derived computationally using the quotient of female subjects count over total participant population. The statistical evaluation revealed that gender (the proportion of females within the sample) did not exert a statistically meaningful moderating influence on the MIL-SI association coefficient [*r* = −0.389, 95% CI (−0.427, −0.349), *p* = 0.708].

Thirty-nine samples were entered into sub-sample analysis (Table 5). A mixed effects model indicated that the correlation between MIL and SI was- 0.391 [95% CI (−0.431, −0.350)] in females and −0.363 [95% CI (−0.495, −0.214)] in males.

**Table 5:** Moderator analysis of gender, economy, tools, and public health emergency.

Moderator variable	Subgroup	K2*	Effect size and 95% interval	*I^2^*	*p*
Gender	Male	8	−0.391 (−0.431, −0.350)	98.12%	0.962
	Female	31	−0.363 (−0.495, −0.214)		
Economy	Developed regions	21	−0.302 (−0.350, −0.255)	99.03%	0.000
	Underdeveloped regions	8	−0.481 (−0.623, −0.339)		
	cross-regional	10	−0.353 (−0.431, −0.276)		
Tool	Chinese-revised	28	−0.387 (−0.433, −0.342)	99.16%	0.000
	foreign original	11	−0.314 (−0.392, −0.235)		
Public health emergency	Before covid-19	25	−0.356 (−0.412, −0.302)	87.65%	0.004
	After Covid-19	14	−0.338 (−0.410, −0.272)		

*K2 = Number of Studies.

#### 3.5.2 Economy

Based on the division of developed and undeveloped regions by Su and his colleagues (2021), 21 samples were conducted in developed regions, eight were conducted in undeveloped regions, and 10 were cross-regional. As indicated in Table 5, the analytical results reveal that the economy exerts a pronounced moderating impact on the MIL-SI association within the Chinese context [*r* = −0.346, 95% CI (−0.385, −0.307), *p* < 0.001].

Analysis of a mixed-effects model revealed that the MIL-SI association measured −0.481 [95% CI (−0.624, −0.339)] in less-developed regions, whereas this value attenuated to −0.303 [95% CI (−0.350, −0.255)] in highly-developed regions. Marked variability in association strengths was observed across less-developed and highly-developed regional groupings. The correlation between MIL and SI, as measured by surveys conducted in undeveloped regions, is stronger than the correlation obtained from surveys conducted in developed regions.

#### 3.5.3 Tool

The SI measurement tools utilized in the studies encompassed by this study were categorized based on the sample reports into two distinct types: Chinese-revised tools and foreign original tools. In sub-sample analysis, 28 samples employed Chinese-revised tools, while 11 utilized foreign original tools. As demonstrated in Table 5, we found that tools significantly moderate the relationship between MIL and SI in the Chinese context [*r* = −0.346, 95%CI (−0.385, −0.307), *p* < 0.001].

A mixed effects model indicated that the correlation between MIL and SI was −0.387 [95% CI (−0.433, −0.342)] in Chinese-revised tools samples, while it was −0.314 [95% CI (−0.392, − 0.235)] in foreign original tools samples. Significant correlation heterogeneity was found in Chinese-revised and original foreign tools samples. The correlation between MIL and SI, as measured by surveys using Chinese-revised tools, is stronger than the correlation obtained from surveys using foreign original tools.

#### 3.5.4 Public health emergency

In sub-sample analysis, 25 samples were conducted before the pandemic, while 14 were conducted after. As demonstrated in Table 5, we found that public health emergency (COVID-19) significantly moderated the relationship between MIL and SI in the Chinese context [*r* = −0.346, 95% CI (−0.385, −0.307), *p* < 0.01].

A mixed-effects model demonstrated that the MIL-SI correlation measured −0.356 [95% CI (−0.412, −0.302)] in pre-pandemic samples, whereas this value declined to −0.338 [95% CI (−0.410, −0.272)] in post-pandemic samples. Significant correlation heterogeneity was found in samples before and after the pandemic. The correlation between MIL and SI, as measured before COVID-19, is stronger than the correlation obtained from surveys after COVID-19.

## 4 Discussion

### 4.1 Main effect of meaning in life and suicidal ideation

To our knowledge, this meta-analysis is the first to date on the associations between MIL and SI in the Chinese context. Analytical findings demonstrated an inverse MIL-SI relationship (*r* = −0.387) among Chinese people, supporting hypothesis 1. A series of studies have demonstrated a negative correlation between MIL and SI. The lower the MIL, the higher the frequency of SI (Liu et al., 2013; Liu and Xie, 2015; Luo et al., 2016).

The significant effect size underscores the critical role of MIL as a psychological buffer against SI in China. Our results suggest that interventions designed to bolster MIL could be particularly impactful in this context. The strength of this association may be amplified by culturally salient factors. The profound emphasis on family responsibility and collective purpose within Chinese society appears to provide potent pathways for individuals to derive meaning. When individuals perceive their lives as contributing to family welfare or broader social cohesion, values deeply embedded in the cultural fabric, it may create a uniquely resilient buffer against despair. This connection is directly reflected in our meta-analytic result. Furthermore, this cultural dimension potentially intensifies the universal protective mechanism of MIL identified in previous research.

While existential theories such as Frankl’s concept of the existential vacuum offer a broad framework for understanding the link between meaninglessness and suicidal ideation (Frankl, 1985), the specific protective mechanism of MIL against SI within China appears distinctly channeled through culturally specific values. Research indicates that in the Chinese context, the experience of an existential vacuum is frequently mitigated through engagement with family roles and social responsibilities (Lin et al., 2022). Confucian principles emphasizing responsibility to family and society provide individuals with transcendent goals that anchor life values and reduce suicide risk associated with existential emptiness (Zhang et al., 2016). Crucially, individual value and MIL are often derived from contributions to the family unit (Zhang et al., 2011b), and meaning is intrinsically tied to family and societal harmony within the collectivist orientation (Ding, 2023). Therefore, although the core protective function of MIL in combating existential nihilism remains universal, our meta-analysis empirically affirms that its potency as a buffer against SI in the Chinese population is actively shaped and significantly amplified by these specific cultural conduits: familial duty and collective identity.

### 4.2 Moderating effects of the relationship between meaning in life

#### 4.2.1 Gender

The analysis of moderators on the relationship between MIL and SI in the Chinese context demonstrates that there is no significant influence in the relationship between MIL and SI across different genders. Therefore, hypothesis 2 of this study is not supported.

As a crucial psychological resource, MIL can effectively buffer suicidal ideation in both men and women. However, its protective mechanism demonstrates unique cultural adaptability against the backdrop of China’s social gender role transformation.

Firstly, modern societal development in China, alongside the transformation of gender roles, weakens the traditional gender-based segregation in meaning construction. With the acceleration of urbanization, the spread of education, and the rise in the proportion of working women, the traditional social division of labor where “men work outside, women manage inside” has been broken. Research shows that the percentage of urban women in China deriving life meaning from professional achievements has increased by 32% compared to 20 years ago (Wang and Wang, 2023). Meanwhile, men’s increasing participation in childcare and housework has brought them closer to women in terms of deriving meaning from family roles (Zhang D. et al., 2022; Zhang J. et al., 2022). This fluidity in social roles reduces the one-way constraint of traditional gender norms on the sources of meaning.

Secondly, policy-driven gender equality strengthens the cross-gender protective effects of MIL. Policies such as the Anti-Domestic Violence Law (2016) and the revised Women’s Rights Protection Law (2022) provide institutional support to reduce structural pressure differences through systemic intervention. This allows both genders to access social support resources more equally, thereby enhancing the risk-resilience efficacy of MIL.

Finally, the deep cultural resilience of normative meaning construction mediates gender differences. Despite changes in social roles, the core value placed on family by traditional culture continues to profoundly influence both genders. Data indicates that 76% of Chinese men and 82% of women rank “family responsibility” as the primary source of life meaning (Zhang et al., 2011b). This consensus results in the protective mechanisms of MIL exhibiting cross-gender stability.

Moreover, gender differences may indirectly influence the relationship between MIL and SI through mediating variables such as social support and coping strategies, rather than directly moderating this relationship. Although women face greater psychological stress, their stronger social support networks may mitigate risks (Caetano et al., 2013; Shye et al., 1995), while men’s reluctance to seek help might exacerbate the accumulation of stress (Möller-Leimkühler, 2003; Oliffe et al., 2020). However, these pathways may not have been incorporated into the analytical framework.

The heterogeneity in sample size and research methods within the meta-analysis may have diluted the signal of gender differences. Characteristics of the sample (such as a predominant student population), variations in measurement tools, or differences in study design among included studies may obscure actual gender effects, rendering them statistically non-significant in aggregated analyses. Future research should optimize sample representation and refine analytical models to comprehensively elucidate the potential role of gender in the relationship between MIL and SI.

#### 4.2.2 Economy

Preliminary evidence suggests that the relationship between MIL and SI varies depending on economic conditions within the Chinese context. Surveys conducted in undeveloped regions show a stronger correlation between MIL and SI compared to those in developed regions, which supports hypothesis 3.

This finding is similar to the results reported by Li and colleagues. One possible explanation for this result is that people living in undeveloped regions place greater importance on material conditions that improve their quality of life (Batson and Stocks, 2004; McLeod, 2007). Their MIL is associated with the pursuit of these material conditions, forming a clear and enduring sense of life meaning in the process (King and Hicks, 2009; Stephens et al., 2013).

China’s rapid urbanization has created an economic gradient between urban and rural areas, which is actually a spatial imprint of traditional agricultural civilization and industrial civilization (Wang and Hui, 2018). This reshapes the cultural construction logic of MIL, leading to profound spatial heterogeneity in the relationship with SI. In undeveloped regions, Confucian filial piety ethics have been distorted into a materialistic survival competition, residents anchor MIL on rigid material goals (Jeung et al., 2019), tying their MIL to material achievements that improve family economic conditions (Batson and Stocks, 2004), forming a condition-dependent MIL (King and Hicks, 2009). A collapse in the economy can trigger a breakdown in the MIL. In contrast, developed regions experience rapid economic development, and sources of MIL shift toward non-material dimensions such as personal value realization and social participation (Li et al., 2024). However, the “996 work schedule” and the “identity split of new citizens” still create semi-modern dilemmas (Li, 2017). Durkheim’s theory of anomie presents an urban–rural fractal here: the dual dilemma of the disintegration of traditional cultural support networks in rural areas and the absence of new social norms in cities (Durkheim, 2005), making the meaning systems of materially deprived groups more prone to collapse (McLeod, 2007).

#### 4.2.3 Tools

The present meta-analysis showed that the moderating effect of tools on the relationship between MIL and SI was significant and supported hypothesis 4. The samples included in this study utilized SI measurement tools that were either Chinese-revised or original SI measurement tools. The selection of tools has a substantial impact on the study of the correlation between MIL and SI.

This phenomenon can be explained from three perspectives. First, the cross-cultural validity of measurement tools is itself culturally constrained. A typical example is the Western-developed “Depression Symptoms-Suicide Subscale” (DSI-SS), which bundles the measurement of depression and suicidal symptoms based on the theoretical assumption (that suicide is primarily driven by psychological disorders) rooted in the Western medical model. However, the prevalence of psychological disorders in Chinese suicide cases is significantly lower than in the West (Phillips et al., 2002). This core cultural specificity difference means that when DSI-SS is applied to Chinese populations, it may overestimate the explanatory power of depression on Suicidal Ideation (SI) due to cultural mismatch (Zhang et al., 2016). Second, the process of localizing scales is itself a form of cultural adaptation and semantic reconstruction. For instance, the Chinese version of the “Beck Scale for Suicide Ideation” (BSI) deliberately replaces the original term “suicidal intent” with “thoughts of self-harm.” This adjustment in wording is not a simple translation but deeply aligns with the widespread taboo against directly discussing death and the tradition of euphemistic expression in Chinese culture (Xu, 2021), directly reflecting the influence of cultural norms on measurement language. Lastly, there are also differences in the weight of core dimensions for assessing suicide risk across different cultural backgrounds, as evidenced by the focus of scale design: The commonly used Western “Suicidal Behaviors Questionnaire-Revised” (SBQ-R) places more emphasis on observable behavior frequency, whereas the Chinese domestically developed “Self-Rating Idea of Suicide Scale” (SIOSS) focuses more on the authenticity of individual self-reports and inner experiences. This difference reflects how deep cultural value orientations shape the construction logic and core concerns of assessment tools.

#### 4.2.4 Public health emergency

The present study demonstrated that the moderating effect of public health emergency on the relationship between MIL and SI was significant. Moreover, the correlation between MIL and SI, as observed in surveys conducted before COVID-19, exhibits a stronger association compared to that found in surveys conducted after COVID-19, partly supporting hypothesis 5.

This result could be explained from two perspectives. On the one hand, the prolonged nature of the Pandemic has subjected individuals to chronic stress, leading to the gradual depletion of psychological resources (e.g., social support, emotional energy). According to the Conservation of Resources Theory (COR, Hobfoll, 1989), long-term resource loss weakens individuals’ ability to buffer suicide risk through MIL—when individuals fall into an “existential vacuum” (Frankl, 1985), the ambiguity of goal perception and the rupture of value connections may exacerbate SI (Trzebiński et al., 2020). This is reflected in the weakened negative correlation between MIL and SI in post-pandemic samples compared to pre-pandemic cohorts.

On the other hand, in the process of responding to the COVID-19 crisis, the Chinese government has constructed a globally unique path to enhance psychological resilience through institutional meaning provision and the deep activation of collectivism cultural genes. This model is first embodied in the institutional practice of the governance philosophy of “people first” – universal free vaccination, material supply systems, and precise prevention and control measures. By providing “security provision,” the protective power of the state is transformed into an existential anchor point for citizens’ “national community identity,” gaining extremely high support for epidemic prevention policies (Zhai, 2023). Secondly, relying on social mobilization advantages, community volunteer actions transform the ethical norm of “respecting one’s own elders and extending that respect to others’ elders” in Chinese culture into “micro-practices of protecting home and country.” During lockdown management, numerous residents participated in mutual aid, allowing individuals to reconstruct “helping behaviors” as “crisis-driven missions” (Liu et al., 2021; Martela and Steger, 2023). This dynamic balance of resilience wisdom ultimately forms a real-time transformation mechanism of “loss control-meaning creation” (Van Bavel et al., 2020), enabling individuals to maintain the Confucian life realm of “peaceful existence and meaningful life” under pressure. The resulting structural effect is that institutional meaning provision partially replaces the anti-suicide function of individual MIL, leading to an abnormal weakening of the negative correlation between MIL and SI during strict prevention and control periods, highlighting China’s uniqueness in reshaping suicide prevention strategies during the pandemic through cultural factors.

## 5 Conclusion

This meta-analysis establishes a robust negative correlation between meaning in life MIL and suicidal ideation SI in China, confirming MIL’s critical protective role. The relationship is significantly moderated by three contextual factors: it strengthens markedly in economically undeveloped regions, varies by cultural appropriateness of SI measurement tools, and intensifies during public health crises like COVID-19. Gender, however, showed no significant moderating effect. These findings highlight MIL’s context-dependent power as a psychological buffer against SI within China’s sociocultural landscape.

Practically, suicide prevention efforts should prioritize individuals with low MIL—particularly in underserved regions—through targeted psychological support and public health initiatives that cultivate purpose. Culturally sensitive SI assessment tools are essential for accurate risk identification. Post-crisis mental health responses must integrate MIL-building interventions (e.g., logotherapy) alongside physical health monitoring. Future research should investigate the mechanisms linking cultural values (e.g., familial duty) to MIL’s protective effects, develop tailored MIL-enhancement interventions for high-risk groups, and validate context-specific measurement tools across diverse Chinese populations.

## 6 Limitations

This study has three key limitations. First, potential publication bias favoring significant MIL-SI associations may affect effect size estimates despite comprehensive searches. Second, findings are constrained by the overrepresentation of urban-educated populations (e.g., college students), limiting generalizability to rural and low-socioeconomic groups. Third, the cross-sectional nature of included studies prevents causal conclusions about the MIL-SI relationship. Future research should actively recruit underrepresented populations and employ longitudinal designs to establish causality while controlling for confounders like mental health status.
